# Diffuse idiopathic skeletal hyperostosis of cervical spine - An unusual cause of difficult flexible fiber optic intubation

**DOI:** 10.4103/1658-354X.62609

**Published:** 2010

**Authors:** Vaibhavi Baxi, Sucheta Gaiwal

**Affiliations:** Associate Specialist, Department of Anesthesia, Lilavati Hospital and Research Center, Mumbai, India; 1 Consultant, Department of Anesthesia, Lilavati Hospital and Research Center, Mumbai, India

**Keywords:** *Cervical spine*, *DISH*, *difficult intubation*, *fiberoptic intubation*

## Abstract

This is a report of anterior osteophytes on the cervical vertebra resulting in distortion of the airway and leading to difficulty during intubation. The osteophytes associated with the syndrome of diffuse idiopathic skeletal hyperostosis were at the C2-3 and C6-7, T1 level and resulted in anterior displacement of the pharynx and the trachea respectively.

## INTRODUCTION

Diffuse idiopathic skeletal hyperostosis (DISH) is a condition characterized by calcification and ossification of ligaments and enthuses (ligament and tendon insertion sites); mainly affecting the vertebral column. Ossification of the anterior longitudinal ligament is common, may be discontinuous, and is often more marked in the thoracolumbar spine than elsewhere. However, isolated and predominant cervical spinal involvement may occur. DISH occurs primarily in the elderly population and is often associated with the syndromes of osteoarthritis and ossification of the posterior longitudinal ligament (OPLL). Radiological evaluation may be useful in assessing the airway in patients deemed to be at risk for difficult intubation. Careful clinical evaluation of the airway before operation and having an approach to the anticipated difficult intubation are emphasized.

## CASE REPORT

**Figure 1:**
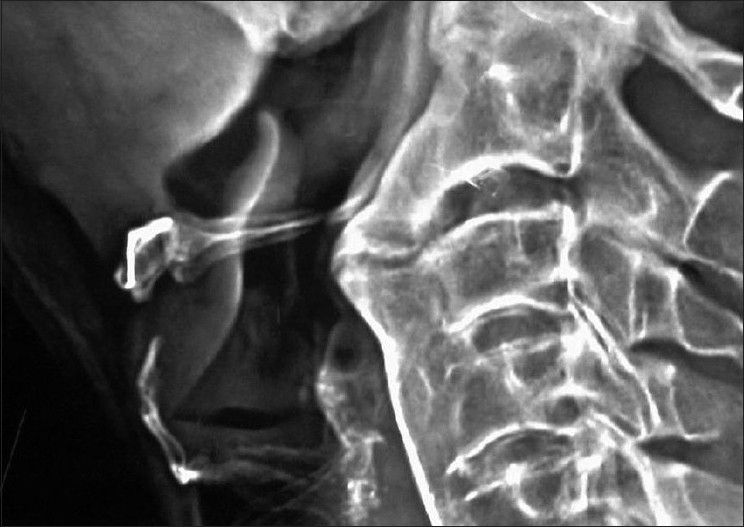
Lateral x-ray of cervical spine

**Figure 2:**
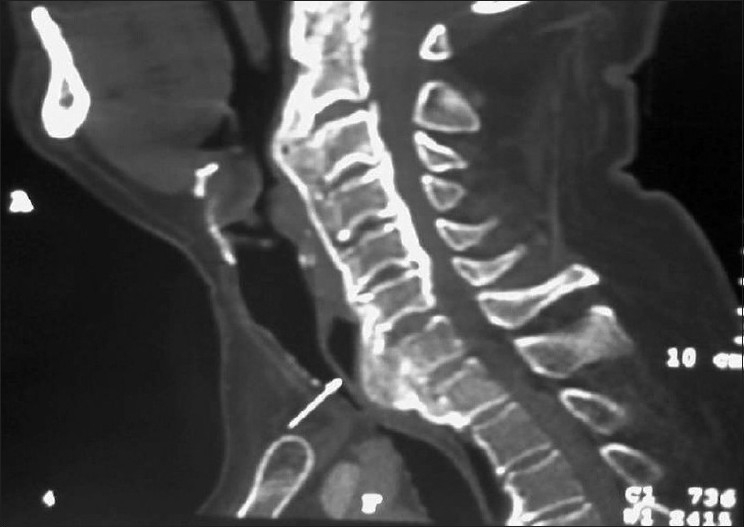
MRI of cervical spine

A 54-year-old male weighing 90 kg and diagnosed with DISH was scheduled for elective anterior decompression and plating at cervical spine level C2-3 and C6-7 level. He was a chronic smoker and smoked about 10-15 cigarettes a day. He came with one-month history of progressive weakness and numbness of the lower extremities. He had mild dysphagia for several years and during the six months before admission, the dysphagia had worsened. There was no history of dyspnea or stridor. Radiographs of the cervical spine and magnetic resonance images (MRI) of the neck were obtained. The lateral radiograph of the cervical spine [Figure 1] revealed normal bone density and presence of new bone formation at C2-3 and C6-7, T1 vertebrae with anterior fusion. There were large "crowbeak" shaped oteophytes protruding anteriorly and impinging upon the posterior wall of pharynx at C2-3 level causing narrowing at the level of osteophytes. Magnetic resonance imaging showed neural foraminal narrowing from C2-C3 and C6-C7, with mild canal stenosis. The radiologist reported the presence of anterior osteophyte formation on the vertebral bodies of C2-3 and C67, T1 with anterior displacement of the laryngopharynx and trachea respectively [Figure 2]. Examination of the airway revealed easy visualization of the soft palate, fauces, uvula and pillars (Mallampati class I). Flexion and extension of the cervical spine were normal. Difficult intubation was anticipated in view of the radiological findings and following discussion about anesthetic choices and intubating techniques, awake fiber optic intubation was planned.

The patient had accepted the suggestion of awake intubation and informed consent was obtained. The patient was continually monitored through a sphygmomanometer, pulse oximeter and electrocardiograph. Topical anesthesia of the oropharynx was achieved with 10% lidocaine spray. The patient was preoxygenated while 1 mg midazolam and 50 mcg fentanyl were slowly given intravenously for sedation. The patient was somnolent but easily arousable and able to follow commands. His respiratory rate was about 12 breaths/minute; oxygen saturation greater than 95% on room air. Glycopyrrolate was administered preoperatively to decrease oral secretions. Local anesthesia of the upper airway was accomplished with topical lidocaine and superior laryngeal nerve and transtracheal blocks. At the initial attempt at oral fiber optic laryngoscopy using the Williams airway, neither the epiglottis nor the vocal cords could be identified because of the expected presence of a large soft tissue mass extending from the posterior pharyngeal wall to the base of the tongue. The presence of large anterior cervical osteophyte prevented endotracheal intubation due to significant mass obstruction. With patient′s cooperation and repeated attempts at fiber optic bronchoscopy the epiglottis was noted to be deviated to the left of the midline, and the vocal cords could only be visualized briefly with deep inspiration. Intubation was difficult but accomplished successfully with 8.0 flexometallic cuffed endotracheal tube lubricated with lidocaine gel. Narrowing of tracheal lumen, presumably at the level of C6-7 was observed by the fiberoptic bronchoscope but endotracheal tube bypassed the narrowed segment without any difficulty. Anesthesia was maintained with intermittent doses of Atracurium and Sevoflurane in oxygen: air mixture (50:50). Surgery proceeded uneventfully. Total time for anesthesia and surgery was 45 and 180 minutes respectively.

In view of the difficult intubation, extubation was planned over a Cook^®^ airway exchange Catheter. The muscle relaxation was reversed with neostigmine 0.05 mg/kg and glycopyrrolate 0.006 mg/kg. The patient however became dyspnoeic with obstructive pattern and tracheal tug. Oxygen saturation was although maintained up to 90-92% with oxygen supplement by the cook′s catheter. Airway edema was suspected and patient was re-intubated over the cook′s catheter and shifted to intensive care unit with stable hemodynamics, for ventilator support. Patient was extubated after 36 hours postoperatively over a fiber optic bronchoscope. Rest of the postoperative period was uneventful. Patient was discharged from the intensive care area and hospital after three and seven days respectively.

## DISCUSSION

DISH, also known as Forestier disease, is disorder of unknown origin. It was first described by Forestier and Rotes-Querol in 1950.[1] It is characterized by diffuse ossification and calcification of tendons, ligaments, and fasciae in both the axial and the appendicular skeleton. This disease has a predilection for men (65%) and is common in patients over the age of 50, with a prevalence of approximately 15-20% in the elderly population.[2,3] The spinal column is most often affected with the thoracic spine being most commonly involved (95% of the patients), followed by the lumbar and cervical spines.[4,5]

According to Resnick,[6] the radiographic diagnostic criteria in the spine include: 1) osseous bridging along the anterolateral aspect of at least four vertebral bodies; 2) relative sparing of intervertebral disc heights, with minimal or absent disc degeneration; and 3) absence of apophyseal joint ankylosis and sacroiliac sclerosis.

The clinical manifestations are variable. Some subjects are completely asymptomatic, while others complain of back pain and stiffness and symptoms secondary to encroachment of the bony excrescences on neighboring structures. Pharyngoesophageal and tracheal compression may result in dysphagia, dyspnea, and stridor.[7] The reported incidence of dysphagia in patients with DISH ranges from 0.2-28%; dyspnea is less common.[8] Myelopathy associated with OPLL is another possible complication since OPLL has been described in association with DISH in up to 50% of cases.[9] DISH also predisposes to complications such as iatrogenic mucosal and laryngeal damage during endoscopic procedures and intubation.[10] Conventional radiography is usually sufficient to confirm the diagnosis of DISH and dynamic video fluoroscopy should be reserved for demonstrating the exact relationship of the cervical spine alterations with the swallow function in those patients who are experiencing dysphagia.[11] CT and MRI may be used to better detect associated findings (e.g., OPLL) and complications (e.g., spinal canal stenosis and compressive myelomalacia).[5]

Dysphagia in patients with DISH involvement of the cervical spine develops mainly because of mechanical compression causing varying degrees of esophageal obstruction, impaired epiglottic motility, and distortion of the laryngeal cartilages. Moreover, chronic compression may result in an inflammatory process in the esophageal wall that can lead to fibrosis and adhesions, with fixation of the esophagus and disruption of the neural plexus.[8] Alterations of the upper cervical spine, especially at the C3-C4 level, may interfere with laryngeal function,[10] while more distal involvement may induce spasm of the upper esophageal sphincter (which is usually located at C5-C6) or esophageal compression. [8,12]

Anterior bone deposition in the cervical spine in patients with DISH will have variable effects on the anatomy of the airway depending not only on the levels involved but also the amount and orientation of the bone deposited. In our patient, extensive bone deposition was on the second-third cervical level which resulted in anterior displacement of the pharyngolarynx. The osteophyte in present case had grown enough to cause airway distortion leading to difficult intubation as in other reported cases of cervical osteophytes.[13,14] Presence of the large anterior cervical osteophyte prohibited successful visualization of the glottis. Anticipation of difficult intubation due to airway distortion made fiberoptic laryngoscopy to be the logical choice. Elective tracheostomy was not planned in view of prolonged morbidity associated with it. Laryngeal mask airway could have been tried but site of surgery prevented its use.

## CONCLUSION

Osteophytes are likely to be missed in routine airway examination. A high index of suspicion is required to anticipate this condition if there is history of dysphagia, dyspnea, sensation of lump in the throat or change in character of voice in patients with cervical spine disorders. In such cases a lateral radiograph of cervical spine should be considered. Quantification of the airway abnormality can be done by MRI or 3-D computed tomography in doubtful cases.
